# Impact of COVID-19 on the practice of breast pathologists: a survey of breast pathologists in the UK and Ireland

**DOI:** 10.1136/jclinpath-2021-207725

**Published:** 2021-10-07

**Authors:** Mirna Elghobashy, Lutful Wahab, Anu Gunavardhan, Emma O’Sullivan, Elena Provenzano, Rahul Deb, Susan Pritchard, Silvana Di Palma, Ian O Ellis, Clinton Boyd, Sarah E Pinder, Abeer M Shaaban

**Affiliations:** 1 University of Birmingham, College of Medical and Dental Sciences, Birmingham, UK; 2 Histopathology, West Hertfordshire Hospitals NHS Trust, Watford, UK; 3 Histopathology, Betsi Cadwaladr University Health Board, Bangor, UK; 4 Public Health England, London, UK; 5 Pathology Department, Addenbrooke's Hospital, Cambridge, UK; 6 Cellular Pathology, University Hospitals of Derby and Burton NHS Foundation Trust, Derby, UK; 7 Histopathology, Wythenshawe Hospital, Manchester, UK; 8 Histology, Royal Surrey County Hospital NHS Foundation Trust, Guildford, UK; 9 Molecular Medical Sciences, University of Nottingham, Nottingham, UK; 10 Histopathology, Belfast Health and Social Care Trust, Belfast, UK; 11 Academic Oncology/Breast Pathology, King's College London, London, UK; 12 Cancer and Genomic Sciences, University of Birmingham, Birmingham, UK; 13 Cellular Pathology, Queen Elizabeth Hospital, Birmingham, UK

**Keywords:** breast, COVID-19, pathology department, hospital

## Abstract

**Aims:**

There is little information on the impact of COVID-19 on breast pathologists. This survey assessed the effect of the COVID-19 pandemic on UK and Ireland-based breast pathologists to optimise working environments and ensure preparedness for potential future pandemics.

**Methods:**

A 35-question survey during the first wave of COVID-19 infections in the UK including questions on workload, working practices, professional development, training, health and safety and well-being was distributed to consultant breast pathologists and responses collected anonymously.

**Results:**

There were 135 responses from breast pathologists based in the UK and Ireland. Most participants (75.6%) stated that their workload had decreased and their productivity dropped. 86/135 (63.7%) were given the option of working from home and 36% of those who did reported improved efficiency. Multidisciplinary team meetings largely moved to virtual platforms (77.8%) with fewer members present (41.5%). Online education, including webinars and courses, was utilised by 92.6%. 16.3% of pathologists reported shortages of masks, visors or gowns as the the most common health and safety concern. COVID-19 had a significant negative impact on the physical and mental health of 33.3% of respondents. A small number of pathologists (10.4%) were redeployed and/or retrained.

**Conclusion:**

The UK and Ireland breast pathologists adapted to the rapid change and maintained service delivery despite the significant impact of the pandemic on their working practices and mental health. It is important to apply flexible working patterns and environments that improve productivity and well-being. The changes suggested should be considered for long-term shaping of breast pathology services.

## Introduction

SARS-CoV-2 was first discovered in Wuhan, China, in late 2019. Since then, it has taken on its infamous name ‘COVID-19’ and spread across the world, causing significant impact on all aspects of healthcare.^1^

During the first wave of infections in the UK, the breast screening programme was paused, and emergency surgical and pathology guidelines were produced. These were quickly adopted to prioritise surgery for poor prognosis breast cancers and offer bridging endocrine therapy for the less aggressive, hormone receptor positive tumours.^2^ Histopathologists in general, and breast pathologists in particular, have therefore had to rapidly adapt to the changes in breast cancer care algorithms and their impact on pathology laboratories.^3^

The Royal College of Pathologists issued guidelines which stated that digital reporting may be used to hasten the reporting of urgent cases and to maintain pathology services during the pandemic.^4^ How widely this has been implemented in the context of breast reporting is not clear, particularly since Public Health England is yet to endorse digital pathology for reporting breast screening samples.

The temporary pausing of breast screening in March 2020 has led to a backlog of patients awaiting screening or treatment for breast cancer, which was estimated to affect almost a million women.^5 6^ Breast pathologists, therefore, faced unprecedented service challenges, the full effects of which on their workload, working practices and well-being remain unknown. These are all critical factors in determining our ability to retain the workforce and respond to ongoing or future pandemics.

Therefore, the UK National Coordinating Group for Breast Pathology (NCCBP) in collaboration with the Association of Breast Pathologists (ABP) surveyed practising breast pathologists in the UK and Ireland to understand the impact of the COVID-19 pandemic on pathologists and breast screening practice and the preparedness of various units to ongoing and potential future pressures.

## Method

A 35-question anonymised survey, including quantitative and qualitative questions, was devised to assess, in depth, the impact of the COVID-19 infection first wave on breast pathologists. JISC, an online UK-based survey tool used for academic research, and which is General Data Protection Regulations (GDPR) compliant, was used. Ethical approval was not required as this study did not involve human or animal subjects. The themes explored in the survey included the following:

- Working practices
- Health and safety
- Effect on continuing professional development (CPD)/learning.
- Effect on second opinions
- Effect on research
- Effect on training
- Effect on pathologists’ well-being
- Practices going forward (learning points)

The survey was initially trialled by pathologists on the NHS Breast Screening Programme (NHSBSP) NCCBP to fine-tune any technical/practical issues before opening it to the wider breast pathologist community. In order to promote it to the target audience, the survey was distributed to the emailing list of the NHSBSP pathologists who participate in the UK Breast External Quality Assurance Scheme, the ABP and delegates of national breast pathology update course emailing lists. A 6-week window was given for completion with a reminder email sent 1 week before the survey deadline.

## Results

A total of 135 responses were received from breast pathologists across the UK and Ireland. The response rate was approximately 20%. The majority of respondents were female (60.7%) and had over 10 years of breast pathology experience (83%). The full demographic data of the participants are summarised in table 1. The main findings are described below.

**Table 1:** Demographics of the survey participants

Demographics		Number of participants	Percentage of participants (%)
Age	<40 years	16	11.9
	40–50 years	52	38.5
	50–60 years	51	37.8
	60–70 years	13	9.6
	>70 years	3	2.2
Sex	Male	49	36.3
	Female	82	60.7
	Prefer not to say	4	3.0
Years of histopathology experience	<1 year	1	0.7
	1–5 years	6	4.4
	5–10 years	16	11.9
	>10 years	112	83.0
Ethnicity	White (British)	52	38.5
	White (any other background)	28	20.7
	Asian	45	33.3
	Black African/Caribbean	3	2.2
	Arab	5	3.7
	Mixed	0	0
	Any other ethnic background	2	1.5
UK region	Scotland	6	4.4
	Northern Ireland	8	5.9
	Wales	14	10.4
	North East	3	2.2
	North West	10	7.4
	Yorkshire and the Humber	17	12.6
	West Midlands	23	17.0
	East Midlands	13	9.6
	South West	5	3.7
	South East	9	6.7
	East of England	15	11.1
	Greater London	12	8.9

### Impact of COVID-19 on working practices

**Figure 1:**
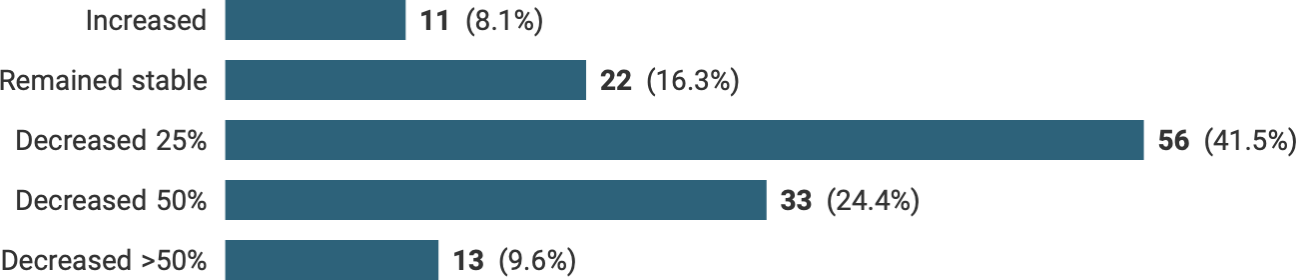
Changes in the workload of breast pathologists during the COVID-19 pandemic.

Respondents were asked about their change in breast workload since the start of the pandemic. One hundred and five out of 135 participants (75.6%) reported that their workload had decreased, with 13/135 stating that their productivity had dropped by over 50%. Conversely, 11 out of 135 (8%) reported that their workload had, in fact, increased during this time (figure 1).

Eighty-six out of 135 (63.7%) of the surveyed pathologists were given the option of working from home. The majority of pathologists did not use it fully, with 39/86 reporting that 20% or less of their work was done remotely. Of those given the option of working remotely, 51/86 (59%) were provided with microscopes to work from home. Only 9.3% of the surveyed pathologists were given the option of reporting digitally. Short text responses indicated centres that were in the process of digital pathology validation and implementation prior to the pandemic were able to adopt it quickly during the first wave. This process would have taken months to few years under normal circumstances.

There were mixed responses regarding pathologists’ efficiency while working from home with 31 (36%) reporting improved efficiency while 24 (27.9%) believed their productivity has decreased.

The majority of respondents reported that there was an increase in virtual meetings rather than face-to-face (77.8%) and less frequent laboratory working (44.4%). Examples were sought, as free-text questions, in order to identify trends in workload changes. These identified that many pathologists felt their working pattern was largely unchanged.

**Figure 2:**
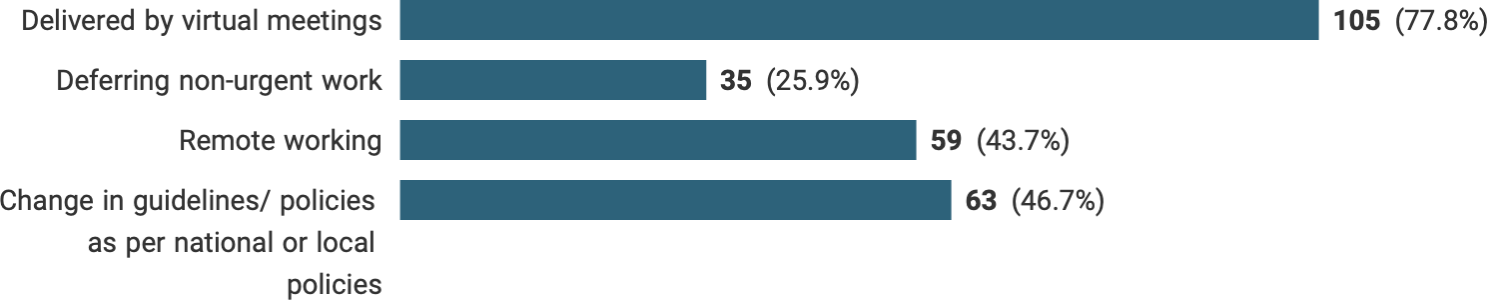
Changes in breast working practices during the COVID-19 pandemic.

A large majority (129/135, 93.3%) of respondents stated that their centre had prioritised patients for surgical or oncological management. This was based on national guidelines (76.2%), local guidelines (49.2%) and following multidisciplinary team meeting (MDTM) decisions (65.1%). Regarding MDTMs, the most common change was virtual meetings (77.8%) and with fewer core members present (41.5%), figure 2.

Cut up of surgical specimens is a vital step in histological diagnosis. Short text responses indicated cut ups progressed as normal on-site. Pathologists working flexibly or from home during the pandemic were required to conduct cut ups in their departments. Participants reported that biomedical scientist (BMS)-led cut ups were helpful when available although this was not found in every department. Short comments also indicated a reduction in frozen section and intraoperative consultation due to the risk of aerosol spread of COVID-19.

During the pandemic, it was necessary to redeploy NHS consultants onto the wards. Of the pathologists surveyed, however, only 14 out of 135 (10.4%) were asked to be redeployed or retrained. For 4 of the 14 pathologists, this redeployment was compulsory. Only 8 out of the 14 redeployed pathologists felt confident working outside their area of expertise.

### Health and safety

**Figure 3:**
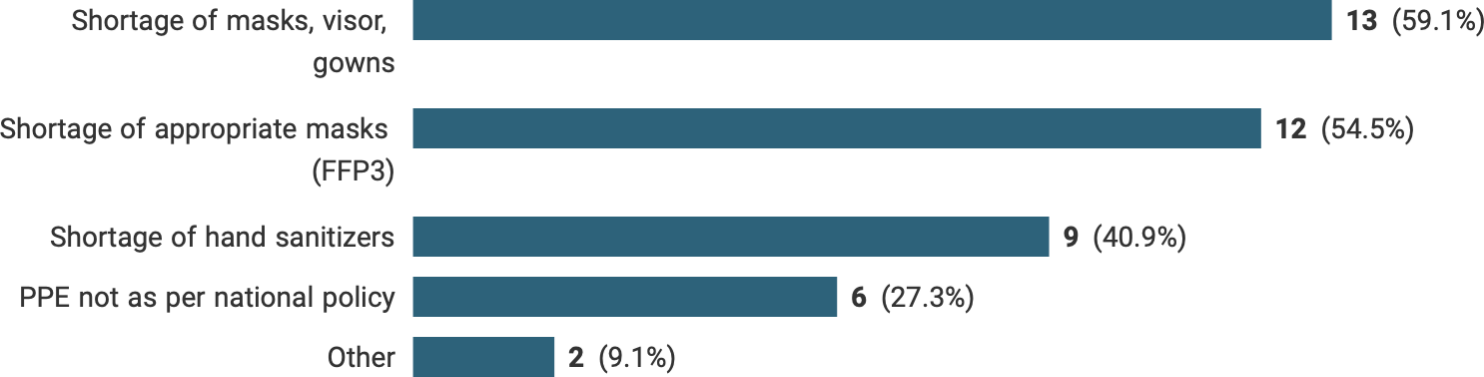
Details of health and safety concerns reported by pathologists.

**Figure 4:**
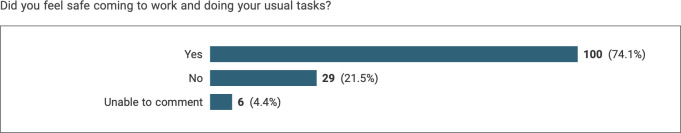
Pathologists’ perception of feeling safe at work.

A total of 37 pathologists (27.4%) reported that they and/or their families had had COVID-19 symptoms and had to self-isolate. Worryingly, 22 out of 135 (16.3%) breast pathologists felt they were not provided with adequate personal protective equipment (PPE); the most common issue was a shortage of standard face masks, visors or gowns (59.1%) followed by a shortage of appropriate masks, such as FFP3 (54.4%). Nine of 22 (40.9%) felt that there was a shortage of hand sanitiser (figure 3). Short text responses identified specific concerns in certain sites such as a slow introduction of mandatory mask-wearing and inadequate laminar air flow to open fresh or unfixed specimens. Additionally, some reported that it was difficult to socially distance while preforming cut ups. Only 90/135 (66.7%) had a formal risk assessment. While most of respondents felt safe coming into work (74.1%), 21.5% felt unsafe performing their daily tasks at work (figure 4).

### Effect on CPD/learning

CPD is vital to maintain the quality of care to patients and update knowledge, particularly during a challenging time such as a pandemic. Of the 125 (92.6%) breast pathologists who accessed additional online educational material, the most utilised resources were webinars (80%) and virtual courses (84.8%). The general consensus was that these were useful or very useful resources (55.2% and 35.2% respectively).

### Effect on second opinion practice

Second opinion consultations are best practice for confirming diagnoses and guiding management of difficult/challenging cases. Reassuringly, 131 out of 135 (97%) breast pathologists were able to obtain a second opinion when required although 32.1% of respondents experienced delays. Out of those who reported that they were unable to obtain a second opinion on physical glass slides, one respondent stated that they sent photographs for virtual case discussions instead.

### Effect on research

Few respondents reported that they contributed to research activities before the COVID-19 pandemic (n=45) and the majority’s answers (n=90) were not applicable. For the remaining responders who took part in research activities, 64.4% reported a negative impact of COVID-19 with only 20% finding more opportunities for research.

### Effect on pathologists’ training

The continuation of medical training and maintaining standards for education are vital for professional development of trainee grade pathologists. There were mixed responses regarding the effect on trainees as a result of the pandemic; 61 out of 135 (45.2%) respondents stated that there was no change in training, 46 (34.1%) said trainees were given less responsibility and 23 (17%) said trainees were given more responsibility although they were not reporting independently. Fifty out of 135 (37%) stated that they were unable to double-report and provide feedback to trainees as usual. Of those 50, only 28 (58%) had alternative arrangements put in place. These included checking and reporting over virtual applications such as Microsoft Teams and Zoom teaching sessions. However, short text responses indicated that if digital was unavailable or malfunctioning, they were unable to provide training for the trainees.

### Well-being

During the COVID-19 pandemic, 127 out of 135 breast pathologists surveyed felt that they had received adequate information regarding health and well-being from their hospital. Despite this, 33.3% felt that COVID-19 had a significant negative impact on their physical and mental health; issues identified included changes in policies and practice, workload and inadequate senior support. During the pandemic, 74.1% of respondents reported feeling anxious to some extent, with 28.1% identifying as moderately or severely anxious.

Over a quarter of the pathologists (27.4 %) had had to isolate due to their/their family’s COVID-19 symptoms.

The responses on work–life balance were varied; 40.7% respondents reported that their work–life balance improved, 34.1% remained the same and 25.2% worsened. Concerns and anxieties were mostly focused on their family’s health, their own health and childcare provision.

### Practices going forward (learning points)

Short text responses indicated that many pathologists found virtual meetings, remote meetings and working from home worked well, while information technology (IT) issues were the most common problem reported. Seventy-three out of 135 pathologists would be willing to return to pre-COVID-19 working patterns while 37/135 would not. Going forward, the majority of respondents were keen to adopt flexible working hours, virtual MDTMs, virtual education, remote working and digital pathology into their workplace.

Following the temporary pausing of breast screening services in 2020, there has been a backlog of patients awaiting mammographic screening. Seventy-seven out of 135 (57%) respondents felt that their departments were not well prepared for dealing with the potential surge of workload due to this backlog. Notwithstanding this, 69.6% said that their department would be prepared for a second wave.

## Discussion

To our knowledge, this survey is the first of its kind in the current literature to provide real life evidence of the changes and challenges reported by breast pathologists during the COVID-19 pandemic.

The aim was to assess the impact of the COVID-19 pandemic on relevant domains of breast pathologists’ practice, health and well-being in order to optimise the working environment, which will subsequently have a positive impact on patient outcomes.

There was a general consensus that workload overall decreased. This was in line with Gathani *et al* who found that there was a decrease in breast referrals made in the first 6 months of 2020, although not as much as was previously feared.^5^ It was suggested that the general decrease in workload was in line with the suspension of breast screening services. However, it does not explain the unexpected finding of some pathologists experiencing an increase in workload during the pandemic. This may be the result of the gradual increase in the screen-detected and symptomatic patients seen and managed by UK breast units from week 4 onwards following the initial pause.^7^ It might also be the result of redistribution of the surgical work, with surgeons in some (but not all) centres having the opportunity to operate in central/COVID-19-free environments, leading to redistribution of the pathology work between hospitals. It is also possible that there was redistribution in work between pathologists in the same department, with some working from home and others in the hospital. Recent data from 10 UK breast units confirmed a change in practices, with standard management applied in between 25% and 59% and the frequency of bridging endocrine therapy ranging from 2% to 35% across the contributing units.^7^ The authors reported a decrease in both screen-detected and symptomatic patients from 16 March 2020 (week 1) with gradual increase from week 4 onwards. Adaptation through prioritisation and new patient pathways were also implemented in other cancer types such as gynaecologic cancer.^8^

It is very likely that pathologists will feel the effects of the backlog in cancer care in the UK and Ireland and they will experience increased workloads with the resumption of breast screening. Indeed, at the time of writing, the national backlog in the UK Breast Screening Programme is approximately 1 050 000 women.^9^ This figure includes both high-risk and routine screening women on BS Select and NBSS database (personal communication from Public Health England, Screening). Using microsimulation models, it has been suggested that delays in breast cancer screening, without screening catching up, would lead to an increase in the cancer death rate by 2 in 100 000 in 10 years. Delayed screening but with immediately catching up screening appointments minimised this effect but required an expansion of screening capacity.^10^ This latter seems optimistic, given the difficulty in fully staffing screening units even before the pandemic.

This backlog will inevitably create a burden on clinical services and, with a consequence increase in diagnostic and therapeutic specimens, on pathology laboratories across the UK. It has never been more important to adopt efficient and creative ways of working that ensure high-quality service delivery and good turnaround times while maintaining the currently stretched pathology workforce. We hope that findings from this survey will assist in the development of short-term coping strategies in pathology laboratories but also future long-term strategic planning. Data from the current survey, however, shows that over half (57%) of the respondents believe their pathology departments are not prepared to cope with the expected surge of activities.

Similar cancer workload trends have been seen across Europe. A single institution audit of cancer detection rates in one of the worst COVID-19-infected province in Italy showed a reduction of cancer diagnosis in 2020 by 39% compared with the previous year. A moderate reduction (26%) in breast cancer diagnosis was found. The sharpest fall in diagnosis was seen however in prostate, bladder and colorectal cancers (75%, 66% and 62% respectively).^11^

One of the adaptations that helped pathologists maintain diagnostic services during the COVID-19 pandemic was the use of digital pathology platforms. The pandemic has been a catalyst for departments to implement this technology both for onsite and home working. A recent survey of digital trends during the pandemic found that 6/18 of the units found digital pathology reporting from home eased the work force crises and 7/18 stating that it maintained appropriate turnaround times for specimen reporting.^12^ The UK Royal College of Pathologists has been proactive; it provided an emergency response with guidance for departments on home working risk assessment and information on equipment specifications and display.^4^ Similar initiatives and recommendations were produced in Europe to try to maintain high-quality service and turnaround times during the pandemic.^13^ In addition to its use in daily diagnostic practice, digital pathology also allowed effective communication and enhanced consultations, discussion and education among pathologists, which were all critical during this challenging time.^14^

While much pathology reporting can be done remotely, certain tasks, such as specimen cut up, can only be done on site. Investment in, and expansion of, an appropriately trained workforce, such as advanced practitioner/BMS cut up, would relieve pressure on the falling number of trained histopathologists and enable remote reporting.

There were mixed responses from participants regarding productivity at home. This may be due to subjective environmental and personal factors. It would seem appropriate to explore further, and at local level, the reasons for the reported increase in productivity while working from home. This would potentially not only allow increased efficiency of reporting but assist in staff retention and decrease the footprint of departments as well as avoiding unnecessary exposure to COVID-19 (or subsequent waves or new pandemics) in the workplace. Clearly high-quality microscopes, or monitors for whole slide image digital reporting, would be needed at the pathologist’s home. It was disappointing that many pathology laboratories were slow to adapt and were unable to provide their pathologists with solutions to enable home reporting.

Health and safety is an integral part of the workplace and, therefore, hospitals should ensure that their employees have an appropriate risk assessment. Shortages in the PPE for healthcare professionals were highlighted during the COVID-19 pandemic.^15^ Although pathologists are not front-line workers, mask shortages and a lack of PPE during the pandemic are unacceptable and risk negatively affecting staff physical and mental health and their productivity.

Access to second opinions was largely unaffected during the COVID-19 pandemic; the vast majority of pathologists surveyed were able to access these as required. Posting slides and blocks internationally for expert opinion, for example, was hampered during the first phase of the pandemic. One option to address delays associated with need for physical submission of material for second opinions (as encountered by 32.1% pathologists) is to use digital pathology. This allows pathologists anywhere off-site to assess sections and both local colleagues and expert pathologists to view cases and provide an opinion. Alternatively, as one participant has been using, virtual case discussions may also streamline the process.

While a significant proportion of respondents reported that normal trainee activities were affected due to the pandemic, the long-term impact on training is not known. It will be interesting to see how increasing use of digital pathology reporting platforms, as well as digital and online educational resources, affects trainee pathologists. Although there is much uncertainty over the likely impact of these systems on training and examinations, they may have the potential to mitigate some of the concerns over education raised by the survey.

The survey assessed the impact of COVID-19 on the mental health and well-being of pathologists, since poor mental health can lead to burnout and significant impact on medical professionals’ productivity.^16^ Our data showed that breast pathologists were generally resilient during a physically and mentally challenging time. Pathologists were supported by the rapid responses conducted by the NCCBP to the demands of the pandemic and guidance to pathologists on specimen handling.^17^ This, in conjunction with other national guidance, helped to prioritise patients and maintain MDTMs and service delivery. The adaptations observed in implementing new working patterns, digital pathology for some and virtual MDTMs delivery was remarkable and should be capitalised on going forward.

Our survey is somewhat limited by its number of respondents. Although a response rate of 20% and 135 participants is considered reasonable, these results may not represent the views of all pathologists and units around the UK and Ireland. Additionally, it was necessary to strike a balance between the number of survey questions and details against the practicality and length of the survey to encourage participation. Thus, only the most important questions were selected for inclusion in the survey. Further surveys could aim to investigate more specific aspects of breast pathology, such as the impact of COVID-19 on training or digital pathology during the pandemic.

In conclusion, our data show a number of extremely positive changes that were swiftly implemented during the COVID-19 pandemic and can be taken forward and extended to the post-pandemic times. These include flexible working practices, remote reporting, use of digital pathology, virtual MDTMs and virtual platforms for education.

**Take home messages**

- The UK and Ireland breast pathologists adapted to the rapid change and maintained service delivery despite the significant impact of the pandemic on their working practices and mental health.
- It is important to adopt efficient and creative ways of working that ensure high-quality service delivery and good turnaround times while maintaining the currently stretched pathology workforce.
- Flexible working practices, remote reporting, use of digital pathology, virtual multidisciplinary team meetings and virtual platforms for education can be considered long-term to improve productivity and well-being.

## Data Availability

Data are available upon reasonable request. Full details of the anonymous responses are held by the authors on behalf of the National Coordinating Group for Breast Screening upon reasonable request.

## References

[1] Li H , Liu S-M , Yu X-H , et al . Coronavirus disease 2019 (COVID-19): current status and future perspectives. Int J Antimicrob Agents 2020;55:105951. 10.1016/j.ijantimicag.2020.105951 32234466 PMC7139247

[2] FSSA: Federation of surgical Specialities association . Clinical guide to surgical prioritisation during the coronavirus pandemic, 2020. Available: https://www.rcseng.ac.uk/coronavirus/surgical-prioritisation-guidance/

[3] Bracey T , Arif S , Ralte AM , et al . Histopathology during the COVID-19 pandemic: resilience through adaptation and innovation. Diagn Histopathol 2021;27:108–15. 10.1016/j.mpdhp.2020.12.003 PMC7772581 33391394

[4] Williams BJ , Brettle D , Aslam M , et al . Guidance for remote reporting of digital pathology slides during periods of exceptional service pressure: an emergency response from the UK Royal College of pathologists. J Pathol Inform 2020;11:12. 10.4103/jpi.jpi_23_20 32477618 PMC7245343

[5] Gathani T , Clayton G , MacInnes E , et al . The COVID-19 pandemic and impact on breast cancer diagnoses: what happened in England in the first half of 2020. Br J Cancer 2021;124:710–2. 10.1038/s41416-020-01182-z 33250510 PMC7884714

[6] Freer PE . The impact of the COVID-19 pandemic on breast imaging. Radiol Clin North Am 2021;59:1–11. 10.1016/j.rcl.2020.09.008 33222992 PMC7508539

[7] Dave RV , Kim B , Courtney A , et al . Breast cancer management pathways during the COVID-19 pandemic: outcomes from the UK 'Alert Level 4' phase of the B-MaP-C study. Br J Cancer 2021;124:1785–94. 10.1038/s41416-020-01234-4 33767422 PMC7993073

[8] O'Neill D , El-Ghobashy A . Impact of COVID-19 on gynaecological oncology; a global perspective. Heliyon 2021;7:e06658. 10.1016/j.heliyon.2021.e06658 33829116 PMC8015395

[9] Breast screening and coronavirus: up to 1 million women miss their mammogram: breast cancer now, 2020. Available: https://breastcancernow.org/about-us/news-personal-stories/breast-screening-coronavirus-1-million-women-miss-their-mammogram2021

[10] Kregting LM , Kaljouw S , de Jonge L , et al . Effects of cancer screening restart strategies after COVID-19 disruption. Br J Cancer 2021;124:1516–23. 10.1038/s41416-021-01261-9 33723386 PMC7957464

[11] De Vincentiis L , Carr RA , Mariani MP , et al . Cancer diagnostic rates during the 2020 'lockdown', due to COVID-19 pandemic, compared with the 2018-2019: an audit study from cellular pathology. J Clin Pathol 2021;74:187–9. 10.1136/jclinpath-2020-206833 32561524

[12] Browning L , Fryer E , Roskell D , et al . Role of digital pathology in diagnostic histopathology in the response to COVID-19: results from a survey of experience in a UK tertiary referral hospital. J Clin Pathol 2021;74:129–32. 10.1136/jclinpath-2020-206786 32616541 PMC7841475

[13] Giaretto S , Renne SL , Rahal D , et al . Digital pathology during the COVID-19 outbreak in Italy: survey study. J Med Internet Res 2021;23:e24266. 10.2196/24266 33503002 PMC7901595

[14] Gosney JR , Hofman P , Troncone G , et al . Cellular pathology in the COVID-19 era: a European perspective on maintaining quality and safety. J Clin Pathol 2021;74:64–6. 10.1136/jclinpath-2020-206789 32482888 PMC7299655

[15] O'Sullivan ED . PPE guidance for covid-19: be honest about resource shortages. BMJ 2020;369:m1507. 10.1136/bmj.m1507 32303504

[16] Rajgopal T . Mental well-being at the workplace. Indian J Occup Environ Med 2010;14:63–5. 10.4103/0019-5278.75691 21461156 PMC3062016

[17] NCCBP ABS COVID-19 guidelines. guidances in relation to breast pathology services, 2020. National coordinating group for breast pathology and association of breast surgeons. Available: https://associationofbreastsurgery.org.uk/for-members/covid-19-resources/

